# H2AK119Ub1 and H3K27Me3 in molecular staging for survival prediction of patients with pancreatic ductal adenocarcinoma

**DOI:** 10.18632/oncotarget.2126

**Published:** 2014-06-22

**Authors:** Shi Chen, Jiangzhi Chen, Qian Zhan, Yi Zhu, Hao Chen, Xiaxing Deng, Zhaoyuan Hou, Baiyong Shen, Yanling Chen, Chenghong Peng

**Affiliations:** ^1^ Department of Surgery, Shanghai Institute of Digestive Surgery, Ruijin Hospital, Shanghai Jiaotong University School of Medicine, Shanghai, China; ^2^ Department of Hepatobiliary Surgery, Union Hospital, Fujian Institute of Hepatobiliary Surgery, Fujian Medical University, Fuzhou, China; ^3^ Department of Hepatobiliary Surgery, Fujian Provincial Hospital, Fujian Medical University, Fuzhou, China; ^4^ Department of Biochemistry and Molecular Cell Biology, Shanghai Key Laboratory for Tumor Microenvironment and Inflammation, Shanghai Jiaotong University School of Medicine, Shanghai, China

**Keywords:** histone modification, molecular staging, prognosis, pancreatic cancer

## Abstract

Polycomb group (PcG) proteins Ring1B and EZH2, which have been characterized as catalyzing the two epigenetic modifications H2AK119 monoubiquitination (H2AK119Ub1) and H3K27 trimethylation (H3K27Me3), are well-known epigenetic silencers implicated in embryonic development and tumorigenesis. However, the status of polycomb-associated histone modifications and their clinical implications in pancreatic cancer remain unclear. Here, we performed immunohistochemistry on tissue microarrays (TMAs) containing 80 pairs of human pancreatic cancer specimens to assess the expression levels of Ring1B, H2AK119Ub1, EZH2, and H3K27Me3 in tumors. More than 50% of the tumor cells showed a high expression of H2AK119Ub1, Ring1B, and EZH2, whereas more than 50% of the tumor cells showed a low level of H3K27Me3. Different expression patterns of H2AK119Ub1 and H3K27Me3 in tumors were negatively correlated (r = −0.247, P = 0.027). Both H2AK119Ub1 and H3K27Me3 independently predicted the clinical prognosis. In particular, a combinatorial pattern of elevated H2AK119Ub1 and decreased H3K27Me3 in tumors was significantly correlated with a poorer prognosis. Furthermore, compared to the tumor, lymph node, metastasis (TNM) staging system, histone modifications can discriminate the survival difference more accurately, especially for patients with stage I or stage II tumors. Simultaneous silencing of Ring1B and EZH2 via shRNA depleted H2AK119Ub1 and H3K27Me3 in the pancreatic cancer cells PanC1 and AsPC1, enhanced HOX gene derepression, and inhibited tumor cell growth in vitro and in tumor xenograft models. These results demonstrated that H2AK119Ub1 and H3K27Me3 cooperate in tumors and are associated with the clinical prognosis in combinatorial patterns. We have proposed that epigenetic modifications may serve as discriminatory biomarkers for molecular staging of pancreatic cancer.

## INTRODUCTION

Pancreatic ductal adenocarcinoma (PDAC) is one of the deadliest human malignancies, with a 5-year survival rate of only 5% and a median survival of less than 6 months[1]. Characterized as being extremely aggressive and highly chemo-resistant, this disease remains a health challenge that is far beyond our understanding of the molecular mechanisms fueling tumorigenesis. It has been thought that epigenetic events are involved in tumorigenesis, including DNA methylations, post-translational histone modifications, and chromatin remodeling[2-4]. Global histone modifications supply independent prognostic information for several tumor types, including prostate[5], gastric[6], lung[7], and breast tumors[8]. Therefore, unraveling the epigenetic code alterations in PDAC may provide information for molecular staging and potential antitumor targets.

Two well-known histone modifications are H2AK119 monoubiquitination (H2AK119Ub1) and H3K27 trimethylation (H3K27Me3), both of which are mediated by polycomb group (PcG) complexes. PcG complexes were initially identified to be involved in the developmental regulation of drosophila, and they are epigenetic gene silencers that can repress transcription of HOX genes (27). Ring1A and Ring1B, core components of polycomb repressive complex 1 (PRC1), are responsible for H2AK119Ub1; while EZH2 and SUZ12, core components of polycomb repressive complex 2 (PRC2), are responsible for H3K27Me3[9]. Recent studies have shown that EZH2 and Ring1B are upregulated in many cancers and highly correlate with a poor prognosis[10-12]. In addition, aberrant expression of H3K27Me3 found in tumor is correlated with a poor outcome[13, 14]. However, whether global histone modifications mediated by PRC1 and PRC2 cooperate in the crosstalk networks during tumorigenesis and correlate with the clinical prognosis remains elusive.

The concept of molecular staging, which may help to distinguish tumor subtypes from molecularly heterogeneous and different prognosis, has been proposed and investigated in several human cancers[15, 16]. Here, we show that both elevated H2AK119Ub1 and decreased H3K27Me3 are significant independent predictors for a poor clinical prognosis. The combinatorial pattern of elevated H2AK119Ub1 and decreased H3K27Me3 in tumors was significantly correlated with a poorer prognosis, especially for patients with stage I or stage II tumors. Combinatorial silencing of Ring1B and EZH2 depleted H2AK119Ub1 and H3K27Me3 in pancreatic cancer cells, enhanced HOX gene derepression, and inhibited tumor cell growth both *in vitro* and *in vivo*, indicating the potential link between H2AK119Ub1 and H3K27Me3. These results suggest that polycomb-associated histone modifications may serve as biomarkers for molecular staging of pancreatic cancer to discriminate subgroups of patients with more aggressive tumors and a poorer prognosis.

## RESULTS

### Different expression patterns of H2AK119Ub1 and H3K27Me3 in PDAC

**Figure 1:**
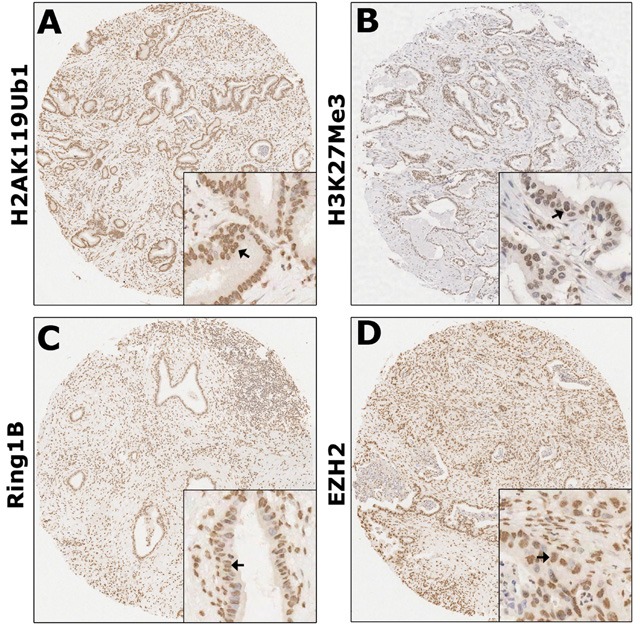
Global levels of histone modifications in PDAC were detected by immunohistochemistry Immunohistochemical detections of H2AK119Ub1 (A), H3K27Me3 (B), Ring1B (C), and EZH2 (D) were performed on TMAs of human PDAC specimens. Arrows indicate positively stained cells. Both H2AK119Ub1 and H3K27Me3 were nucleic proteins; both Ring1B and EZH2 were expressed predominantly in the nuclei. Original magnification: ×40.

**Figure 2:**
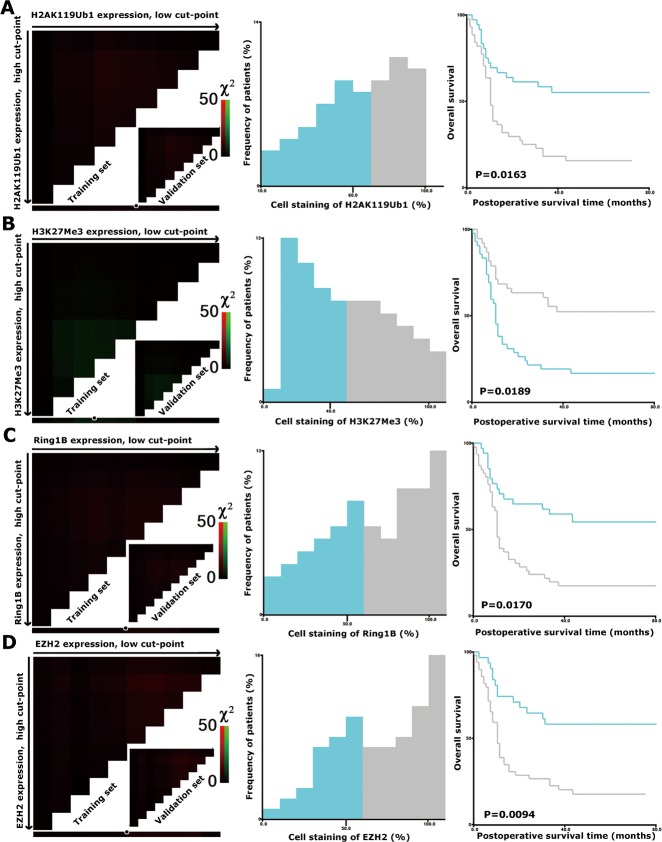
X-tile plots of H2AK119Ub1, H3K27Me3, Ring1B, and EZH2 on PDAC TMAs X-tile analysis was performed on a cohort of 80 cases of pancreatic cancer, which were equally divided into a training subset and a validation subset. X-tile plots of training sets are shown in the left panels, with matched validation sets in the inset. The plot displays the χ^2^ log-rank values. The black/white circles in the left panels highlight the cutoff points, which are shown on a histogram of the whole cohort (middle panels) and a Kaplan-Meier plot (right panels). P values were calculated by using the cutoff point derived from the training subset to create a separate validation subset. The expression levels of H2AK119Ub1 (A), H3K27Me3 (B), Ring1B (C), and EZH2 (D) were divided at the optimal cutoff points as defined on the plot. The optimal cutoff points for the four markers were as follows: ≤60% and >60% of tumor cells with positive staining of H2AK119Ub1; ≤50% and >50% of tumor cells with positive staining of Ring1B or EZH2; ≤40% and >40% of tumor cells with positive staining of H3K27Me3; P < 0.05 indicated statistical significance.

High-throughput analysis of the four markers in tumors was performed by immunohistochemical detection on tissue microarrays (TMAs) containing 80 PDAC tumorous specimens. The staining patterns of H2AK119Ub1, H3K27Me3, Ring1B, and EZH2 were predominantly in the nucleus (Figure 1). In order to select the optimal cutoff point, the X-tile program was used to determine cutoff scores for histone modification levels. According to X-tile, the TMA cohort was randomly divided into a training set and a validation set (Figure 2; left panels). The optimal cutoff point determined by the training set was reassessed in the validation set. Depending on the cutoff point, the cohort was divided into low and high populations (Figure 2; middle panels). More than 60% of the cells positively stained for H2AK119Ub1, 40% for H3K27Me3, and 50% for Ring1B and EZH2 were considered to be highly stained. High staining of the tumor cells for H2AK119Ub1, EZH2, and Ring1B was found in most samples (55%, 60%, and 56% respectively). In contrast, only 48% of the tumor samples displayed high staining for H3K27Me3, while 53% of the tumor samples displayed low H3K27 methylation. High expression of H2AK119Ub1, Ring1B, and EZH2 correlated with a short survival time, while low H3K27Me3 expression predicted a poor prognosis (Figure 2; right panels). These data indicated that the expression patterns of individual histone modifications in tumors differ considerably.

### Global levels of histone modifications are associated with tumor malignant behavior

The correlations between the expression levels of histone modifications and clinical characteristics were further examined (Table 2). There were no significant associations between global histone modifications and clinical characteristics including age, sex, tumor location, extent of invasion, perineural invasion, and tumor stage. However, the high H2AK119Ub1 expression group was significantly associated with the PDAC group with a larger tumor size (P = 0.017), poorer differentiation (P = 0.024), and lymph node metastasis (P = 0.016). In contrast, the low H3K27Me3 expression group was significantly associated with the PDAC group, which showed a larger tumor size (P = 0.015), poorer differentiation (P = 0.011), and lymph node metastasis (P = 0.020). These data indicated that individual histone modifications are associated with tumor malignant behavior.

**Table 1:** Patient Characteristics. Clinical characteristics (age, sex) and pathological characteristics (tumor size, location, pathological grade, lymph node metastasis, depth of invasion, venous invasion, perineural invasion, and TNM stage) of PDAC patients are summarized. Tumor staging was evaluated according to the American Joint Committee on Cancer classification system (TNM). Others indicate that the pathological grade was undefined

Characteristic	No. of patients (N=80)	Percentage (%)
Sex Men women	50 30	62.5 37.5
Age Mean ± SD, year	61±11	
Tumor location Head, neck Body, tail	48 32	60 40
Tumor size ≤2 cm >2 cm	35 45	43.8 56.2
Pathological grade Well, moderate Poor, others	54 26	67.5 32.5
Depth of invasion T1, T2 T3, T4	64 16	80 20
Lymph node metastasis Negative Positive	46 34	57.5 42.5
Perineural invasion Negative Positive	46 34	57.5 42.5
Stage I, II III, IV	78 2	97.5 2.5

**Table 2:** Relationship between the expression levels of histone modifications in a tumor and the clinical features. Clinical characteristics of PDAC patients with a high level or a low level of H2AK119Ub1 and H3K27Me3 were tested statistically. Measurement data shown as mean ± SD were analyzed by the independent Student's t-test, and enumeration data were analyzed by the Chi-squared test. N = 80; * indicates statistical significance, P < 0.05; a indicates that the P value was obtained from Fisher's exact test

	H2AK119Ub1		P	H3K27Me3		P	Ring1B		P	EZH2		P
	Low	High		Low	High		Low	High		Low	High	
Sex Male Female	23 13	27 17	0.816	24 18	26 12	0.298	23 11	27 19	0.411	18 13	32 17	0.515
Age <60 years old ≥60 years old	23 13	22 22	0.213	24 18	21 17	0.866	20 14	25 21	0.690	21 10	24 25	0.099
Tumor size ≤2 cm >2 cm	21 15	14 30	0.017*	13 29	22 16	0.015*	17 18	18 28	0.333	12 19	23 26	0.470
Location Head, neck Body, tail	22 14	26 18	0.854	28 14	20 18	0.201	20 14	28 18	0.853	19 12	29 20	0.851
Pathological grade Well, moderate Poor, others	29 7	25 19	0.024*	23 19	31 7	0.011*	25 9	29 17	0.322	19 12	35 12	0.346
depth of invasion T1, T2 T3, T4	27 9	37 7	0.312	32 10	32 6	0.370	26 8	38 8	0.497	23 8	41 8	0.302
Lymph node metastasis Negative Positive	26 10	20 24	0.016*	19 23	27 11	0.020*	20 14	26 20	0.837	20 11	26 23	0.313
Perineural invasion Negative Positive	22 14	24 20	0.555	25 17	21 17	0.700	21 13	25 21	0.507	20 11	26 23	0.313
Stage I, II III, IV	36 0	42 2	0.499a	40 2	38 0	0.495a	33 1	45 1	1.000a	30 1	48 1	1.000a

### Correlation between different expressions of H2AK119Ub1 and H3K27Me3 in PDAC

To further explore the correlation between different expressional patterns of histone modifications in PDAC, Spearman's rank correlation coefficient analysis was performed (Table 3). We observed a negative correlation between H2AK119Ub1 and H3K27Me3 (r = −0.247, P = 0.007), and a positive correlation between EZH2 and Ring1B (r = 323, P = 0.003). There was no significant correlation between H2AK119Ub1 and Ring1B (r = 0.185, P = 0.101); nevertheless, no significant correlation between H3K27Me3 and EZH2 (r = 0.112, P = 0.321) was observed. Taken together, these data indicated that the expressional patterns of H2AK119 ubiquitination and H3K27 methylation are correlated in PDAC.

**Table 3:** Spearman's correlation of the expression levels of histone modifications in tumors. The expression levels of EZH2, Ring1B, H3K27Me3, and H2AK119Ub1 in PDAC specimens were determined by Spearman's correlation analysis. r indicates Spearman's rho value; n = 80; * indicates statistical significance, P < 0.05

Groups	r value	P value
H2AK119Ub1 vs. Ring1B	0.185	0.101
H3K27Me3 vs. EZH2	0.112	0.321
Ring1B vs. EZH2	0.323	0.003*
H2AK119Ub1 vs. H3K27Me3	−0.247	0.027*

### H2AK119Ub1 and H3K27Me3 in molecular staging for prognosis prediction of PDAC patients

Next, univariate survival analysis corroborated that high H2AK119Ub1, high Ring1B, or high EZH2 expression in PDAC predicted poor survival independently (both P < 0.05; Table 4). When adjusted for tumor size, pathological grade, extent of invasion, perineural invasion, and tumor stage, multivariate analysis showed that high H2AK119Ub1 expression independently predicted a poor survival (HR = 2.051, P = 0.028). Also, high levels of Ring1B or EZH2 were independently correlated with a poor prognosis (HR = 2.419, P = 0.006; HR = 3.530, P < 0.001). In contrast, both univariate and multivariate survival analyses showed that a high level of H3K27Me3 in PDAC was found to have a “favorable” effect on the clinical outcome (HR = 0.480, P = 0.033).

**Table 4:** Multivariate proportional hazard analysis. Relative risk of death was evaluated by Cox regression modelling and global histone modification patterns in PDAC. Multivariate analysis was adjusted for tumor size, pathological grade, lymph node metastasis, perineural invasion, and tumor staging. HR, hazard ratio; CI, confidence interval; n = 80; * indicates statistical significance, P < 0.05

Variables	n	Univariate		P value	Multivariate		P value
		HR	95%CI		HR	95%CI	
Sex Male Female	50 30	1 0.830	0.470–1.466	0.521			
Age (years) <60 ≥60	45 35	1 1.352	0.788–2.320	0.274			
Tumor size ≤2 cm >2 cm	35 45	1 2.079	1.174–3.679	0.012*			
Location Head, neck Body, tail	48 32	1 1.145	0.665–1.692	0.625			
Pathological grade Well, moderate Poor, others	54 26	1 2.210	1.275–3.830	0.005*			
Depth of invasion T1, T2 T3, T4	64 16	1 0.914	0.459–1.822	0.799			
Lymph node metastasis Negative Positive	46 34	1 1.633	0.950–2.806	0.076			
Perineural invasion Negative Positive	46 34	1 1.969	1.094–3.546	0.524			
Staging I, II III, IV	78 2	1 2.209	0.535–9.128	0.274			
H2AK119Ub1 Low High	36 44	1 2.657	1.468–4.810	0.001*	1 2.051	1.082–3.888	0.028*
Ring1B Low High	34 46	1 2.725	1.489–4.988	0.001*	1 2.419	1.285–4.552	0.006*
H3K27Me3 Low High	42 38	1 0.383	0.215–0.681	0.001*	1 0.480	0.244–0.944	0.033*
EZH2 Low High	31 49	1 2.987	1.589–5.614	0.001*	1 3.530	1.793–6.951	0.000*

**Figure 3:**
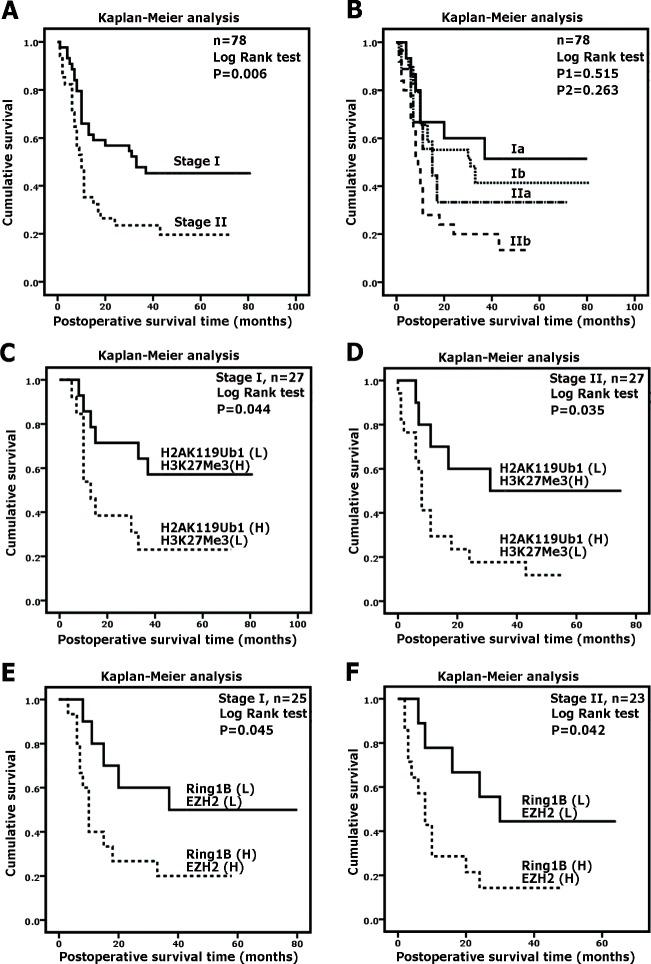
H2AK119Ub1 and H3K27Me3 correlate with clinical prognosis Overall survivals of PDAC patients were analyzed by Kaplan-Meier analysis and the log-rank test. (A) Survival curves for TNM stage I and II. Two patients at stage IV were excluded. (B) Both groups of patients at stage I or stage II were subdivided according to the TNM staging system. P1 was calculated using the log-rank test by comparing the stage Ia group and the stage Ib group; P2 was calculated using the log-rank test by comparing the stage IIa group and the stage IIb group. (C) Stage I or stage II PDAC patients were subdivided as follows: group 1, H2AK119Ub1 (high) and H3K27Me3 (low); group 2, H2AK119Ub1 (low) and H3K27Me3 (high). For stage I or stage II tumors, patients showing high H2AK119Ub1 expression and low H3K27Me3 expression were associated with a poorer survival. (B) Stage I or stage II PDAC patients were subdivided as follows: group 1, Ring1B (high) and EZH2 (high); group 2, Ring1B (low) and EZH2 (low). For stage I or stage II tumors, patients showing high Ring1B expression and high EZH2 expression were associated with a poorer survival.

The overall postoperative survival time was further analyzed by a Kaplan-Meier plot according to the TNM staging system, which is commonly used in clinicopathological staging. Compared with the stage I group, the stage II group was associated with a shorter survival time (Figure 3A, < 0.05). However, there were no significant differences between the survival times of the stage Ia and Ib groups or the stage IIa and IIb groups (Figure 3B, both P > 0.05). Here, based on the correlated expression of H2AK119Ub1 and H3K27Me3 in a tumor, we further determined whether combinatorial patterns of histone modifications predict a poorer clinical prognosis in pancreatic cancer. Thus, we subdivided patients at the same TNM stage according to the immunohistochemical scores. Unexpectedly, a combinatorial pattern of high H2AK119Ub1 expression and low H3K27Me3 expression was associated with an especially poor survival for both stage I and stage II patients (Figure 3C, D; both P < 0.05). Likewise, high EZH2 expression accompanied with high Ring1B expression was correlated with a shorter survival time for patients at TNM stage I or II (Figure 3E, F). Here, we demonstrated that histone modification profiles can separate good and poor prognosis patients better than TNM staging, indicating that H2AK119Ub1 and H3K27Me3 can serve in molecular staging for pancreatic cancer.

### Simultaneous silencing of Ring1B and EZH2 lead to increased HOX gene derepression in pancreatic cancer cells

**Figure 4:**
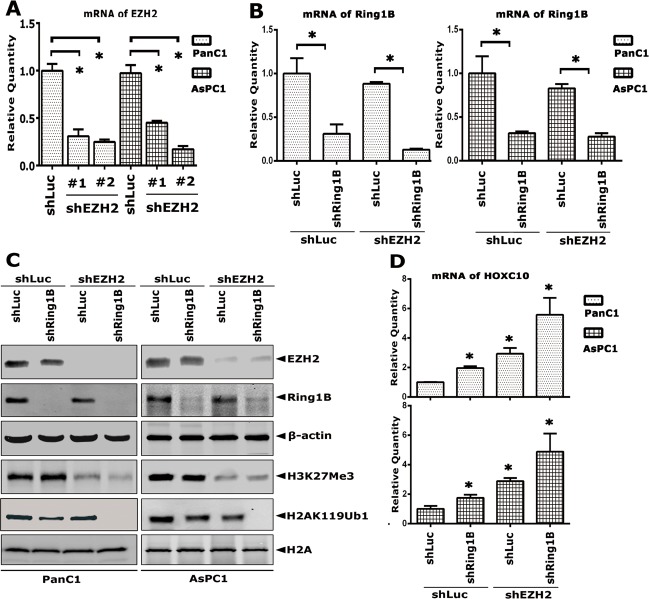
Simultaneous silencing of Ring1B and EZH2 lead to increased HOX gene derepression in pancreatic cancer cells EZH2 was knocked down in PanC1 and AsPC1 cells by specific shRNAs. The mRNA level of the EZH2 gene was analyzed by qRT-PCR. #1 and #2 indicate different cell pools generated from different EZH2 shRNAs. (B) Ring1B was knocked down subsequently in shEZH2-PanC1 and -AsPC1 cells by specific shRNA. The mRNA level of the Ring1B gene was analyzed by qRT-PCR. GAPDH was used as a loading reference. Data were presented as mean ± standard error of the mean (SEM) from three independent experiments. * indicates statistical significance, P < 0.05. (C) Whole cell lysate (100 μg) extracted from shLuc/shEZH2+ or shLuc/shRing1B-PanC1 or -AsPC1 cells were loaded. A total of 3 μg of histone extract for detecting H2AK119Ub1 and H3K27Me3, and 10 μg for detecting H2A were loaded. β-actin and H2A were used as loading controls. (D) The mRNA level of the *HOXC10* gene was analyzed by qRT-PCR. Data were presented as mean ± SEM from three independent experiments. * indicates statistical significance, P < 0.05.

To further confirm the pathological correlation of global histone modifications from the perspective of biological function, EZH2 was knocked down by shRNA in the pancreatic cancer cells PanC1 and AsPC1. Both real-time polymerase chain reaction (qRT-PCR) and western blot analyses showed that EZH2 was effectively depleted in PanC1 and AsPC1 cells (Figure 4A and C, respectively). As a result, the level of H3K27Me3 was sharply reduced accordingly (Figure 4C). Next, Ring1B was knocked down by shRNA in shLuc/shEZH2-PanC1 and -AsPC1 cells subsequently, the results of which were confirmed both by the mRNA and protein level (Figure 4B and C, respectively). H2AK119 monoubiquitination was slightly impaired in the absence of Ring1B or EZH2 independently, while completely depleted in the simultaneous knock down of Ring1B and EZH2. As is well-known, PcG complexes are involved in transcriptional repression of HOX genes epigenetically. Next, the mRNA level of *HOXC10*, which is the target gene of EZH2 and Ring1B, was checked by qRT-PCR assays. Silencing of Ring1B or EZH2 independently caused a slight derepression of *HOXC10*, while simultaneous silencing of Ring1B and EZH2 led to sharp derepression of *HOXC10* (Figure 4D). These data showed that the combined silencing of Ring1B and EZH2 led to increased HOX gene derepression in pancreatic cancer cells.

### Simultaneous depletion of Ring1B and EZH2 lead to inhibition of cell proliferation and tumor growth

**Figure 5:**
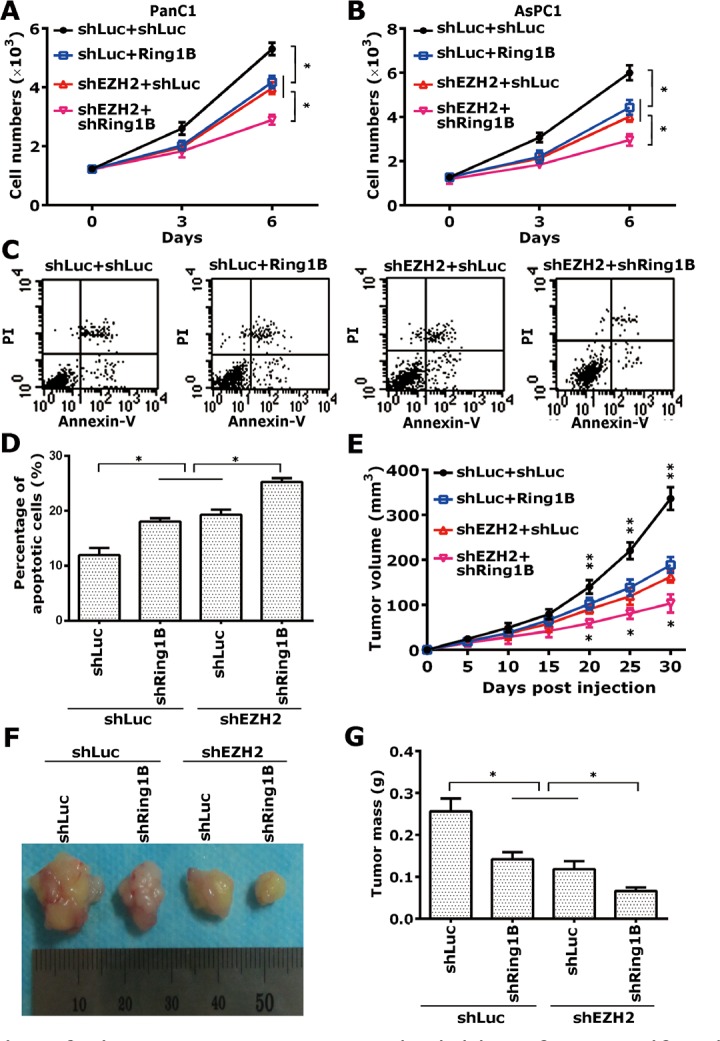
Simultaneous depletion of Ring1B and EZH2 lead to inhibition of cell proliferation and tumor growth (A, B) Equal numbers of shLuc/shEZH2+shLuc/shRing1B-PanC1 and -AsPC1 cells (1 × 10^3^) were plated into 12-well plates, and the cell numbers were subsequently counted at the indicated time points (Days 0, 3, and 6). Data were presented as mean ± standard deviation (SD) from three independent experiments. * indicates statistical significance, P < 0.05. (C) Equal numbers of shLuc/shEZH2+shLuc/shRing1B-PanC1 cells (2 × 10^6^) were plated into 6-well plates. The apoptotic cells were determined using annexin V-FITC conjugated to PI by flow cytometry. The apoptosis analyses shown are representative of three independent experiments. (D) Total numbers of early and late apoptotic cells are shown as histograms. Data were presented as mean ± SD from three independent experiments. * indicates statistical significance, P < 0.05. (E) Equal numbers of shLuc/shEZH2+shLuc/shRing1B-PanC1 cells (4 × 10^6^) were subcutaneously implanted into each mouse. Tumor size was measured every 5 days, and tumor growth was followed for 30 days after tumor cell injection. ** indicates that the shLuc+shLuc group was statistically different compared with the shLuc+shEZH2 or shLuc+shRing1B group, P < 0.05; *indicates that the shEZH2+Ring1B group was statistically different compared with the shLuc+shEZH2 or shLuc+shRing1B group, P < 0.05. Tumor size data were presented as mean ± SD from five independent mice. (F) Representative pictures of the tumors corresponding to each group at day 30 after tumor cell injection are shown. The ruler shows the widths of the tumors in cm. (G) Tumor weight for the indicated groups was measured after the mice were sacrificed. Column data were presented as mean ± SD from five independent mice. * indicates statistical significance, P < 0.05.

To further assess the phenotype of the Ring1B- and EZH2-knocked down pancreatic cancer cells, we performed a cell proliferation assay to determine whether Ring1B and EZH2 are essential for tumor cell proliferation *in vitro*. Equal cell numbers of each clone from shLuc/shEZH2+shLuc/shRing1B- PanC1 and AsPC1 were plated into 12-well plates. After attaching to a dish for 24 h, the total cell numbers of each well were counted at the indicated time. Either Ring1B or EZH2 knock down independently inhibited cell proliferation of pancreatic cancer cells, and simultaneous silencing of Ring1B and EZH2 increased the inhibition of cell proliferation (Figure 5A, B). Next, a cell apoptosis assay was performed to detect apoptotic cells. Ring1B or EZH2 depletion promoted cell apoptosis in PanC1 cells, while codepletion of Ring1B and EZH2 increased the number of apoptotic cells (Figure 5C, D).

Since we established that Ring1B and EZH2 are required for pancreatic cancer cell proliferation *in vitro*, a nude mice-based subcutaneous xenograft model was performed next to investigate the *in vivo* activity of Ring1B and EZH2 on the tumor growth of PanC1 cells. Either Ring1B or EZH2 knock down independently inhibited tumor growth of pancreatic cancer cells, and simultaneous silencing of Ring1B and EZH2 increased the inhibition ability (Figure 5E, F). These data showed that combinatorial silencing of Ring1B and EZH2 inhibited cell proliferation and tumor growth of pancreatic cancer.

## DISCUSSION

Covalent histone modifications, including acetylation, methylation, and ubiquitination on lysine residues, are well-known as “histone codes”, which decode during chromatin remodeling and transcriptional activities. Two well-known histone modifications, H2AK119Ub1 and H3K27Me3, mediated by PcG proteins, are pivotal for normal embryogenesis and cell identity[17, 18], and have been recently reported to be epigenetically altered in human cancers[19, 20]. However, to the best of our knowledge, no studies have investigated the combination of H2AK119 ubiquitination and H3K27 methylation and their potential impact on PDAC tumorigenesis. Here, we demonstrated that high H2AK119Ub1 expression combined with low H3K27Me3 expression in a tumor predicted a poorer prognosis and that elevated Ring1B combined with upregulated EZH2 was associated with a shorter survival time of pancreatic cancer patients, especially for those who were unable to be distinguished by the TNM staging system. These results may shed a new light on molecular staging for pancreatic cancer based on the well-known PcG proteins and epigenetic modifications.

Both PRC1 and PRC2 are involved in transcriptional repression by establishing and recognizing histone modifications during embryonic development and adult tumorigenesis. Recent studies have indicated that Ring1B and EZH2 are required and dysregulated in several types of human cancer[10-12, 21, 22]. In this study, we employed the X-tile program to select the optimal cutoff points and found that more than 50% of tumor cells showed high expression of Ring1B and EZH2. Either a high level of Ring1B or EZH2 predicted a shorter survival time for the PDAC patients. It is noteworthy that there was a positive correlation between the expression patterns of Ring1B and EZH2, and simultaneous silencing of Ring1B and EZH2 led to HOX gene derepression. The mechanisms underlying the correlation between Ring1B and EZH2 are unknown. It has been reported that dynamic repression of developmental pathways by PRC1 and PRC2 may be required simultaneously and that PRC1 and PRC2 coregulate many of the same target loci, such as HOX genes and *ngn1*[23-25]. In addition, PRC1 and PRC2 are equally required in genomic contraction and imprinted repression, indicating their cooperation in higher-order chromatin organization[26]. We also observed that a combination of elevated Ring1B and upregulated EZH2 expression levels was associated with a shorter survival time and that simultaneous knock down of Ring1B and EZH2 promoted apoptosis and inhibited proliferation. These results suggested that a combination of Ring1B and EZH2 may help to distinguish the tumor subtypes, thus providing different prognoses for pancreatic cancer patients.

Strikingly, we found that a high H2AK119Ub1expression and a low H3K27Me3 expression were observed in tumors and correlated with a poor survival for PDAC patients. Simultaneous depletion of H2AK119Ub1 and H3K27Me3 not only led to increased HOX gene derepression but also synergistically inhibited cell proliferation, indicating the cooperation between histone modifications. A previous study has reported that combinatorial patterns of global histone modifications may act cooperatively to prepare chromatin for transcriptional activation[27]. Therefore, it is plausible that PRC2- and PRC1-mediated histone modifications may be subject to some identical pathways during tumorigenesis. In our observation, a high level of H2AK119Ub1 and a low level of H3K27Me3 in tumors were associated with malignant behaviors and predicted a poorer survival time. These results indicated that histone ubiquitination and methylation may serve as biomarkers for molecular staging of pancreatic cancer and can be used to discriminate subgroups of patients with more aggressive tumors and thus a poorer prognosis.

H2AK119Ub1 was slightly impaired due to loss of H3K27Me3 in pancreatic cancer cells, indicating that global H2A ubiquitination was not completely dependent on H3K27 methylation. It is conceivable that H3K27Me3 may be responsible for the fundamental level of H2AK119 ubiquitination; however, there are several other E3 ligases implicated in H2A ubiquitination and these ligases do not depend on H3K27Me3[28]. Furthermore, H2AK119 ubiquitination can be fueled by PRC1 and other E3 ligases, the result of which may lead to hindering H3K27 methylation. Therefore, it will be interesting to reveal the mechanistic timing during different histone modifications, which may unravel the epigenetic crosstalk during transcriptional regulation in cancer.

For pancreatic cancer, it is known that there are subgroups of patients characterized with molecularly heterogeneous and different clinical prognosis. Therefore, it is crucial to determine distinctive molecular biomarkers to discriminate subgroups of patients and direct individual therapeutic intervention. Our study provides evidence that H2AK119Ub1 and H3K27Me3 cooperate in tumors and that the combinatorial expressions of these two histone modifications predict a poorer clinical prognosis. We first propose that epigenetic modifications may serve as discriminatory biomarkers for molecular staging of pancreatic cancer. These results may enlighten the understanding of the epigenetic mechanisms involved in tumors and also merit the investigation of global histone modification in pancreatic cancers.

## MATERIALS AND METHODS

### Tissue microarrays and immunohistochemistry

This study was approved by the Ethics and Research Committees of Shanghai Jiaotong University School of Medicine and was conducted in accordance with the Declaration of Helsinki Principles. Tissue microarrays (TMAs) purchased from ShGnghGi Outdo Biotech Company (China) were well-documented with clinicopathological information, including patient age, sex, tumor size and location, pathological grade, perineural invasion, lymph node metastasis, and tumor staging (detailed in Table 1), as well as follow-up data. Ninety pancreatic cancer patients included in the TMAs underwent pancreatectomy between September 2004 and December 2008, and postoperative follow-up was finished by December 2011. Ten cases without complete follow-up data were excluded. The average follow-up time of the eighty patients was 25.3 months (median, 14 months; range, 1–81 months). Microarrays consisting of one core, each 1.5 mm in diameter, were prepared by dot-arraying tissues from resected human PDAC tumors. Immunohistochemistry (IHC) staining was performed with specific antibodies according to the previous descriptions[29, 30]. EZH2 (Millipore, #15217-662); Ring1B (#5694), H2A (#2578), ubiquityl-Histone H2A (Lys119) (#8240), and Tri-Methyl-histone H3 (Lys27) (#9733) antibodies were purchased from Cell Signaling Technology Inc.

### Evaluation of immunohistochemistry staining reaction

TMAs were scanned by an Aperio Scanscope XT, and the whole field of each dot was obtained for immunohistochemical evaluation. All specimens were strictly evaluated by a senior pathologist, and only those with a tumor content of more than 90% were considered as tumor samples. Only tumor cells were scored, and normal pancreatic cells or infiltrating inflammatory cells were excluded. Scoring of TMA staining was performed by two pathologists blind to the patient clinical information. Semiquantitative assessment of IHC staining for the TMAs was performed according to a previous description[31]. The cells stained as brown nuclei were considered positively stained, and their percentage within the whole tumor tissue was determined. The frequency of positively stained tumor cells (range, 0–100%) was scored for each TMA spot.

### Selection of cutoff point score

The X-tile program (Yale University School of Medicine, version 3.6.1) was used to optimize the cutoff points for assessment of each protein expression according to a previous description[32]. In order to obtain the optimal cutoff points, the cohorts were randomly divided into matched training and validation subsets by X-tile plot. Next, a standard log-rank test was performed to assess the statistical significance by utilizing the cutoff score derived from a training set to parse a separate validation set, and P values were obtained from a table. The X-tile plot program could determine an optimal cutoff value, while the minimum P statistic was corrected by Miller-Siegmund P-value correction.

### Cell culture and shRNA

PanC1 and AsPC1 were cultured in Dulbecco's modified Eagle's medium supplemented with 10% fetal bovine serum and penicillin-streptomycin. The pLKO.1 shRNAs were purchased from Thermo Scientific. The shRNAs targeting EZH2 were CCGGCAACAAGATGAAGAGCACCAACTC and GAGTTGGTGCTCTTCATCTTGTTGTTTTT. The shRNA targeting Ring1B was ATTGTGCTTGTTGATCCTGGC. The stable shRNA-knocked down cells were constructed by viral infection and selected with puromycin.

### Quantitative RT-PCR analysis

Total RNA was isolated from cells with Trizol reagent (Invitrogen). Next, qRT-PCR was performed on a 7500 Fast Real-time PCR system using SYBR Green agent. The following primers were used: hRing1B, forward 5′-GGCAACAAAGAATGTCCTACC-3′ and reverse 5′- GTCACCATTATCTTCTGCTCCA-3′; hEZH2, forward 5′-CCCTGACCTCTGTCTTA CTTGTGGA-3′ and reverse 5′-ACGTCAGATGGTGCCAGCAATA-3′; hHOXC10, forward 5′-CCTCGCAATGTAACTCCGAACT-3′ and reverse 5′-ACCCCGCAATTGAAGTCACT-3′. All qRT-PCR assays were repeated three times.

### Western blot and antibodies

Cells were lysed in buffer containing 20 mM Tris-HCl (pH 8.0), 150 mM NaCl, 2.5 mM EDTA, 0.5% NP40, 0.1 mM phenylmethanesulfonyl fluoride, and protease inhibitors. The methods used for western blot were previously described[33].

### Cell proliferation assay

For the cell proliferation assays, cells were plated in 12-well plates at an equal density of 1 × 10^3^ cells per well, three wells for each cell line in each of three biological repeats of the experiment. After 24 hours (corresponding to Day 0) for cell attachment to the dish, the whole fields of each well were viewed under a microscope at the indicated time points (Days 0, 3, and 6), and the total numbers of cells in each well were counted.

### Cell apoptosis assay

The apoptotic cells were measured using annexin V-FITC conjugated to propidium iodide (PI) by flow cytometry. A total of 1 × 10^6^ cells were plated in 6-well plates per well. After 24 h, the cells were harvested and double-stained with FITC-annexin V and PI using the FITC Annexin V Apoptosis Detection Kit (BD Biosciences), according to the manufacturer's instructions. The cells were analyzed with a flow cytometer (FACScan®; BD Biosciences) equipped with CellQuest software (BD Biosciences). Cells were discriminated into viable cells, dead cells, early apoptotic cells, and late apoptotic cells. Experiments were performed in triplicate.

### *In vivo* mouse study

All animal experiments were overseen and approved by the Animal Welfare Committee of Shanghai Jiaotong University School of Medicine. Tumor growth ability of shEZH2/shRing1B-PanC1 cells were determined by subcutaneous injection into 5-week-old BALB/c athymic nude mice (nu/nu, Slac Laboratory Animal Co. Ltd., Shanghai, China). Five mice were used in each group. A total of 3 × 10^6^ cells were injected subcutaneously into the left rear or right rear flank per mouse. Mouse weight and tumor volume were measured every five days. The width (W) and length (L) of the tumor were measured using a digital caliper, and the tumor volumes were calculated using the formula V = 1/2 (L×W^2^), where L is the length (longest dimension) and W is the width (shortest dimension). Tumor growth was followed for 1 month after tumor cell injection.

### Statistical analysis

Measurement data were analyzed by the independent Student's *t*-test, and enumeration data were analyzed by the chi-squared test or Fisher's exact test. The correlations between each protein were analyzed by Spearman's rank coefficient. Spearman's rho values were categorized according to Dancey and Reidy's categorization: 0 (zero); 0.1–0.3 (weak); 0.4–0.6 (moderate); 0.7–0.9 (strong); and 1 (perfect); r > 0 indicated a positive correlation, r < 0 indicated a negative correlation[34]. The overall survivals were analyzed by the Kaplan-Meier estimator and tested by the log-rank test. A Cox regression model was used for univariate and multivariate analyses. A P value <0.05 was considered to be statistically significant. IBM SPSS Statistics Version 20 software was used for statistical analysis.
